# Emerging genetic complexity and rare genetic variants in neurodegenerative brain diseases

**DOI:** 10.1186/s13073-021-00878-y

**Published:** 2021-04-14

**Authors:** Federica Perrone, Rita Cacace, Julie van der Zee, Christine Van Broeckhoven

**Affiliations:** 1 Neurodegenerative Brain Diseases Group, VIB Center for Molecular Neurology, Antwerp, Belgium; 2 Department of Biomedical Sciences, University of Antwerp – CDE, Universiteitsplein 1, BE-2610 Antwerp, Belgium

**Keywords:** Neurodegenerative brain diseases, Alzheimer’s disease, Parkinson’s disease, Frontotemporal dementia, Amyotrophic lateral sclerosis, Rare coding variants, Missense mutations, Frameshift mutations, Gene discovery, genetic variants of uncertain significance (VUS), functional research

## Abstract

Knowledge of the molecular etiology of neurodegenerative brain diseases (NBD) has substantially increased over the past three decades. Early genetic studies of NBD families identified rare and highly penetrant deleterious mutations in causal genes that segregate with disease. Large genome-wide association studies uncovered common genetic variants that influenced disease risk. Major developments in next-generation sequencing (NGS) technologies accelerated gene discoveries at an unprecedented rate and revealed novel pathways underlying NBD pathogenesis. NGS technology exposed large numbers of rare genetic variants of uncertain significance (VUS) in coding regions, highlighting the genetic complexity of NBD. Since experimental studies of these coding rare VUS are largely lacking, the potential contributions of VUS to NBD etiology remain unknown. In this review, we summarize novel findings in NBD genetic etiology driven by NGS and the impact of rare VUS on NBD etiology. We consider different mechanisms by which rare VUS can act and influence NBD pathophysiology and discuss why a better understanding of rare VUS is instrumental for deriving novel insights into the molecular complexity and heterogeneity of NBD. New knowledge might open avenues for effective personalized therapies.

## Background

Neurodegenerative brain diseases (NBD) are progressive and irreversible fatal conditions primarily affecting the neurons of the central nervous system (CNS). At the cellular level, NBD are characterized by cytoplasmic or nuclear protein aggregations [1]. Dementia symptoms are typical features of NBD and imply a great burden for patients and caregivers [2]. The most frequent NBD subtypes are Alzheimer’s disease (AD) and Parkinson’s disease (PD), followed by the less frequent frontotemporal dementia (FTD) and amyotrophic lateral sclerosis (ALS). The disease characteristics of the NBD subtypes are summarized in Table 1.

**Table 1:** Main characteristics of NBD subtypes

NBD	Brain location	Pathology	Main symptoms
AD	Tempo-parietal lobes	β-Amyloid, tau	Progressive memory loss, cognitive decline
FTD	Frontotemporal lobe	TDP43, tau, FUS	Behavioral changes, language deficits
PD	Midbrain	α-Synuclein, Lewy bodies	Bradykinesia, muscle rigidity, resting tremor. Dementia features in 30–80%
ALS	Motor cortex, spinal cord	TDP-43	Muscle weakness, impaired voluntary movements. Dementia features in 50%

In the last three decades, linkage studies in extended NBD families with a Mendelian inheritance of NBD identified high-penetrant mutations in causal genes and co-segregation with NBD [3, 4]. Causal genes, routinely tested in medical genetic centers for AD, PD, FTD, and ALS, are listed together with mutation spectrum and inheritance patterin in Table 2.

**Table 2:** Causal genes, mutation spectrum, and mode of inheritance

NBD	Causal gene	Type of mutation*	Inheritance	Reference
AD	Amyloid precursor protein (*APP*)	Missense, gene dosage	Autosomal dominant, recessive	[5]
	Presenilin 1 (*PSEN1*)	Missense, indels	Autosomal dominant	[3]
	Presenilin 2 (*PSEN2*)	Missense, indels	Autosomal dominant de novo	[6]
	Prion protein (*PRNP*)	Missense, indels	Dominant	[7]
PD	α-Synuclein (*SNCA*)	Missense, gene dosage	Autosomal dominant	[4]
	Parkin 2 (*PARK2*)	Missense, gene dosage	Autosomal recessive	[8]
	Leucine-rich repeat kinase 2 (*LRRK2*)	Missense	Autosomal dominant	[9]
FTD	Granulin (*GRN*)	PTC	Autosomal dominant	[10]
	Microtubule-binding protein tau (*MAPT*)	Missense, gene dosage	Autosomal dominant	[11]
ALS	Fused in sarcoma (F*US*)	Missense	Autosomal dominant	[12]
	Cu/Zn superoxide dismutase (*SOD1*)	Missense	Autosomal dominant	[13]
	Transactive response DNA-binding protein (*TARDBP*)	Missense	Autosomal dominant	[14]
FTD and ALS	Chromosome 9 open reading frame 72 (*C9orf72*)	G_4_C_2_ repeat expansions	Autosomal dominant	[15]
	TANK-binding kinase 1 gene (*TBK1*)	PTC	Autosomal dominant	[16–18]
	Valosin-containing protein gene (*VCP*)	Missense	Autosomal dominant	[19]

*Abbrevations: indel, insertion/deletion; PTC, premature termination codon

International genome-wide association studies (GWAS) in large cohorts of NBD patients or healthy individuals identified common variants in novel genes showing significant associations to NBD, but with a modest increase in disease risk [20–23]. GWAS NBD risk genes for AD, PD, FTD, and ALS are listed in Table 3.

**Table 3:** NBD risk genes identified in GWAS

NBD	Risk genes	References
AD	*ABCA7*, *ACE*, *ADAM10*, *ADAMTS1*, *APOE*, *BIN1*, *BCKDK*, *CASS4*, *CD2AP*, *CD33*, *CELF1*, *CLU*, *CR1*, *DSG2*, *EPHA1*, *FERMT2*, *HLA-DRB1*, *HLA-DRB5*, *INPP5D*, *IQCK*, *KAT8CR1*, *MEF2C*, *MS4A6A*, *NME8*, *PICALM*, *PTK2B*, *SLC24A4*, *SORL1*, *WWOX*, *ZCWPW1*	[24–30]
PD	*ACMSD*, *ASXL3*, *BCKDK*, *BRIP1*, *BST1*, *C5orf24*, *CAB39L*, *CCDC62*, *CD19*, *CHRNB1*, *CLCN3*, *CRLS1*, *DDRGK1*, *DGKQ*, *DNAH17*, *DYRK1A*, *FAM171A2*, *FAM47E*, *FAM49B*, *FBRSL1*, *FCGR2A*, *FGF20*, *FYN*, *GAK*, *GBA*, *GBF1*, *GPNMB*, *HIP1*, *HLA-DQB1*, *HLA-DRA*, *HLA-DRB5*, *INPP5F*, *KCNIP3*, *KCNS3*, *KPNA1*, *LAMP*, *LCORL*, *LINC00693*, *MAPT*, *MBNL2*, *MCCC1/3*, *MED12L*, *MEX3C*, *MIPOL1*, *NOD2*, *NUCKS*, *PAM*, *RAB29*, *RAB7L1*, *RAI1*, *RIMS1*, *RIT2*, *RNF141*, *RPS12*, *RPS6KL1*, *SCAF11*, *SCARB2*, *SIPA1L2*, *SNCA*, *SPTSSB*, *SREBF1*, *STBD1*, *STK39*,*STX1B*, *SYT11*, *TMEM163*, *TMEM175*, *TRIM40*, *UBAP2*, *UBTF*, *VAMP4*, *VPS13C*	[22, 31–33]
FTD	*BTNL2*, *C4orf27*, *CTSC*, *DPP6*, *HLA-DRA*, *HLA-DRB5*, *HLA-DQA2*, *IMMP2L*, *IRF2*, *MIR548AP*, *OLFM1*, *RAB38*, *RERG*, *TMEM106B*, *UNC13A*	[21, 34]
ALS	*C21orf2*, *DPP6*, *FGGY*, *ITPR2*, *KIF5A*, *MOBP*, *SARM*, *SCFD1*, *UNC13A*	[23, 35, 36]

The genetic discoveries in NBD, i.e., AD, PD, FTD, and ALS contributed to a better understanding of the biological mechanisms underlying CNS neurodegeneration. But, they were not able to adequately disclose the entire genetic background of these complex NBD disorders [37], since NBD is not yet genetically explained in numerous patients [38]. NGS tools, like whole-exome (WES) or whole-genome sequencing (WGS), played a critical role in understanding the pathogenic mechanisms leading to NBD, due to the identification of various novel genes [39–41]. These studies shed a new light on the specific pathways that contribute to NBD pathophysiology. A non-exhaustive list, includes microglia-mediated pathway in AD [20], mitochondrial dysfunction in PD [39], RNA stress response in ALS [40], and lysosomal disruption in FTD [42].

A challenge of these sequencing technologies is the identification of countless rare variants, specifically variants of uncertain significance (VUS) [43, 44]. For a long time, these rare variants were considered non-contributing genetic background without effect on the NBD disease. Consequently, these VUS were ignored, resulting in a lack of supportive genetic and functional data. The rare VUS can be classified based on their likelihood of pathogenicity by using in silico bioinformatic prediction tools [45], but these predictions are insufficient particularly in the situation of a genetic diagnosis of patients [46].

In this review, we focus on rare variant interpretation in NBD phenotypes such as AD, FTD, PD, and ALS. We describe discoveries from WES and WGS studies, including the novel pathways involved in these disorders. We suggest modes of action and strategies for the interpretation of VUS, providing examples of rare variants in established causal genes and GWAS risk genes. We address the impact of improved understanding of rare variants for patients and families and for therapy development. Finally, we deliberate on the potential of omics technologies in unraveling of the genetic etiology and molecular pathways leading to neurodegeneration.

## WES and WGS reveal novel genes and pathways

WES and WGS are mainly used in NBD genetic research to uncover novel genes and pathways [47]. Hand in hand, specific statistical approaches are developed for accurate data analysis and rare variant identification [47, 48]. These tests, analyzing the contribution of multiple variants across candidate gene(s), have increased the power to detect disease association signals [48], often disclosing the clustering of genes in specific pathways.

Examples of recently discovered pathways by application of NGS include microglia alterations in AD, mitochondrial dysfunction in PD, RNA stress response in ALS, and lysosomal disruption in FTD [20, 40, 42, 49].

In AD, besides amyloid processing [50], it is demonstrated that the microglia-mediated pathway is a crucial contributor to the pathogenesis of AD [20]. Rare variants in the triggering receptor expressed on myeloid cells 2 (*TREM2*) were discovered by WGS, showing an increased risk for developing AD [51]. *TREM2* encodes a receptor expressed in myeloid cells that mediates inflammatory responses. The relevance of TREM2 in brain functioning is highlighted by recessive mutations in *TREM2* causing Nasu Hakola disease, and FTD in some patients [51]. Rare heterozygous *TREM2* mutations that increase the risk for developing disease have been described in AD, FTD, ALS, and PD [51], though their role in disease pathogenesis needs further follow-up. The implication of microglial-mediated inflammation in NBD is confirmed by the identification of rare variants in phospholipase C gamma 2 (*PLCG2*) in AD and ABI family member 3 (*ABI3*) [52].

A wide range of evidence indicates mitochondrial dysfunction and mitophagy as important players in PD pathology [53]. A WES study in autosomal-recessive early-onset PD patients identified rare homozygous or compound heterozygous PTC mutations in the vacuolar protein sorting-associated protein 13C gene (*VPS13C*) [54]. VPS13C belongs to a family of vacuolar sorting proteins that are crucial for vesicular transport. VPS13C depletion in neuronal cells leads to the upregulation of the PTEN-induced kinase 1 (PINK1)/parkin (PARK2) gene-dependent mitophagy, where PINK1 normally accumulates on the mitochondria and recruits parkin to initiate mitophagy in response to mitochondrial dysfunction. Moreover, VPS13C loss is associated with lower mitochondrial membrane potential, mitochondrial fragmentation, and increased respiration rates [54].

In ALS, several disease-related genes encode for RNA-binding proteins that interfere with the formation of stress granules [40]. One gene, coding for cytotoxic granule-associated RNA-binding protein (*TIA1*), was identified by WES in a family with both FTD and ALS patients [55]. Mutations in *TIA1* were previously linked to autosomal dominant Welander distal myopathy [56], a muscular dystrophy disease characterized by TAR DNA-binding protein 43 (TDP-43) brain pathology as present in FTD and ALS. TIA1 RNA-binding protein forms stress granules in the cytoplasm upon cellular stress [40]. Rare *TIA1* mutations, linked to FTD and ALS, alter the biophysical properties of TIA1 promoting nucleation of the stress granules and hindering disassembly as the stress stimulus passes [40]. Specific to FTD is that the lysosomal pathway is involved in the pathogenic events leading to disease [18]. This pathway has a role in the degradation of long-lived proteins. Deficits in this pathway result in protein aggregation and generating toxic protein species and accumulation of dysfunctional organelles [57].

Rare PTC mutations were identified by WES in the TANK-binding kinase 1 gene (*TBK1*) leading to the loss-of-function (LOF) of TBK1 and causing FTD or ALS [58]. *TBK1* codes for a serine/threonine kinase, phosphorylating a wide range of substrates involved in several cellular processes, including autophagy. Substrates of TBK1 are optineurin (OPTN) and p62/sequestosome 1 (SQSTM1), which are autophagy adapters controlling protein degradation by selective autophagy. In both genes (*OPTN* and *SQSTM1*), rare mutations were found associated with FTD or ALS. The valosin-containing protein gene (*VCP*) is another gene contributing to FTD and ALS genetic etiology and is also involved in autophagy, emphasizing the major role of autophagic defects in neurodegeneration [58].

Progresses in NGS technologies drastically improved our knowledge of the multiple pathways involved in NBD, including microglia, mitochondrial dysfunction, RNA stress response, and lysosomal disruption. These NGS-driven gene discoveries have also intensified the identification of various rare variants which have an unclear contribution to disease. Statistical association of novel genes with NBD is insufficient to establish pathogenicity. Causal genes and genes functionally associated with a specific NBD subtype are not obligatory showing a significant statistical enrichment in patients [16], as exposed by the ATPase phospholipid transporting 10B gene (*ATP10B*) [49] and the CYLD Lysine 63 deubiquitinase gene (*CYLD*) [59]. The application of WES or WGS and tailored statistics analyses [47] are fruitful to enhance our understanding of the NBD pathogenesis, when the majority of NBD patients remain genetically unexplained [21].

## Genetic, clinical, and pathological heterogeneity in NBD

NBD are complex diseases with a high degree of heterogeneity at the level of genetics, clinical phenotypes, and brain pathology. Besides the distinguishing clinical symptoms, brain pathologies, causal genes, and pathways, NBD present with substantial clinical, genetic, and pathological overlap [60, 61] which may lead to misdiagnoses of the NBD subtypes and erroneous medical treatment or result in grouping of patients for clinical trials that have different underlying pathologies.

Overlapping symptoms of clinical phenotypes, for example, AD and FTD, can affect the diagnosis of the patient at the initiate stage of disease [60].

Loss of short- and long-term memory and cognitive deficits are the classical characteristics of AD, but some AD patients also present with pronounced behavioral changes reminiscent of FTD [62], highlighting the heterogeneity in a single phenotype [63]. Parkinsonism can be present in both AD and FTD patients [26, 27]. The pathological hallmarks in the autopsy brains of AD patients are aggregated amyloid-beta (Aβ) plaques and hyperphosphorylated tau tangles. Yet, among elderly people with definite AD pathology, up to 90% displayed TDP-43 proteinopathy [64], typical of FTD or ALS. Several genetic studies documented the overlap between NBD subtypes, and examples can be found in the well-known disease genes. In the microtubule (MT)-binding protein tau gene (*MAPT*), the missense mutation p.R406W segregates in families of patients with a clinical AD diagnosis and a brain neuropathological of tauopathy [65], while the *MAPT* p.A152T mutation is a risk modifier in other NBD subtypes including AD and dementia with Lewy bodies (DLB) [66]. Mutations in *PARK2* in familial early-onset PD patients are also observed in sporadic early-onset AD patients [67]. In a Belgian founder pedigree, patients carry a LOF mutation in the progranulin gene (*PGRN*), *GRN* IVS1+5G>C and present at autopsy with TDP-43 type A pathology [68]. Yet, in this extended family, some patient carriers received a clinical diagnosis of PD or AD [68]. In light of heterogeneity in clinical diagnoses, a profound investigation of the presence of rare variants in known causal genes and newly identified genes is of paramount importance to improve differential diagnosis [69].

## Relevance of understanding the role of rare variants

In diagnostic genetic testing, WGS, WES, and gene panels are common tools for the identification of mutations in known NBD genes. Identification of rare variants is moving at a faster pace than functional biological interpretation. The data generated by these NGS technologies comprise large numbers of VUS in established disease genes which can create uncertainties as to whether rare variants contribute to disease. For example, in the known AD genes, 68 coding rare variants were reported in the amyloid precursor protein gene (*APP*), 321 in the presenilin 1 gene (*PSEN1*), and 63 in the presenilin 2 gene (*PSEN2*). Yet, a significant fraction of these rare variants has not been functionally investigated: 32.35% in *APP*, 12.77% in *PSEN1*, and 55.55% in *PSEN2*; percentages were calculated from the data in the Alzforum Database, https://www.alzforum.org/mutations, a repository of published mutations in *APP*, *PSEN1*, *PSEN2*, *MAPT*, and *TREM2*. Knowing the contribution of rare variants to disease etiology is highly valuable for patients and their relatives, even if there are no disease-modifying treatments for NBD [70]. In the case of a family with a pathogenic mutation, information can be provided to relatives about genetic testing. In clinical research, mutation carriers can be included in clinical trials, as in the study “Dominantly Inherited Alzheimer Network Trials Unit (DIAN-TU)” in AD [71]. For clinical trials, the knowledge of the role of rare variants in NBD will become a critical aspect to compose homogeneous patient groups for clinical trials, based on a complete genetic profile [72]. In previous studies, patients shared the same clinical diagnosis but often differed in NBD subtype biasing the trial outcomes [73]. Difficulties in clinical differential diagnosis hampered the grouping of patients for research studies [74]. Stratification of patients can be improved by in-depth knowledge of the clinical, pathological, and genetics that are contributing to the NBD of the patient. Variant interpretation is critical for new therapeutic developments. Large families with a high variability in onset age, asymptomatic carriers of pathogenic mutations, and healthy control individuals with control-specific rare variants can be extremely valuable for uncovering possible modifying cellular mechanisms of neurodegeneration.

Induced pluripotent stem cells (iPSCs) can be generated from symptomatic and asymptomatic carriers of a specific variant and differentiated into specific cell types (e.g., neurons) to investigate the effect of the mutations [75]. Alternatively, genome editing technologies (e.g., CRISPR/Cas) can be applied to introduce specific mutations in iPSCs or to generate isogenic control lines [76]. Experiments on iPSCs expressing different pathogenic mutations in the PD gene, leucine-rich repeat kinase 2 (*LRRK2*), e.g., p.G2019S, showed disrupted mechanisms including aggregation of α-synuclein, mitochondrial transport, and lysosomal autophagy [77]. These iPSCs are also beneficial to measure the resulting cellular phenotypes to allow the identification of new therapeutics [78]. For instance, a library including 1258 pharmaceutical compounds was applied to iPSC-derived AD neurons, measuring the secretion of Aβ species (e.g., toxic Aβ42), as an output. In this way, it was possible to identify potential therapeutic compounds able to reduce Aβ42 levels [78].

Three-dimensional (3D) brain organoids derived from human PSCs (hPSCs) and iPSCs can recapitulate the brain’s 3D cytoarchitectural arrangements and provide new opportunities to explore disease pathogenesis [79]. In a recent study, the classical AD phenotypes were recapitulated in familial AD patient-derived 3D brain organoids obtained from familial early-onset AD patients carrying an *APP* duplication [80]. The application of these methodologies can be extended to VUS to verify their possible implication in pathogenic mechanisms underlying an NBD phenotype. In addition, supplementary omics tools are being developed and will provide new opportunities to enhance our understanding of disease mechanisms and investigate how rare variants contribute to NBD pathogenesis.

## Possible modes of action of rare variants in known genes: the penetrance spectrum

**Fig. 1:**
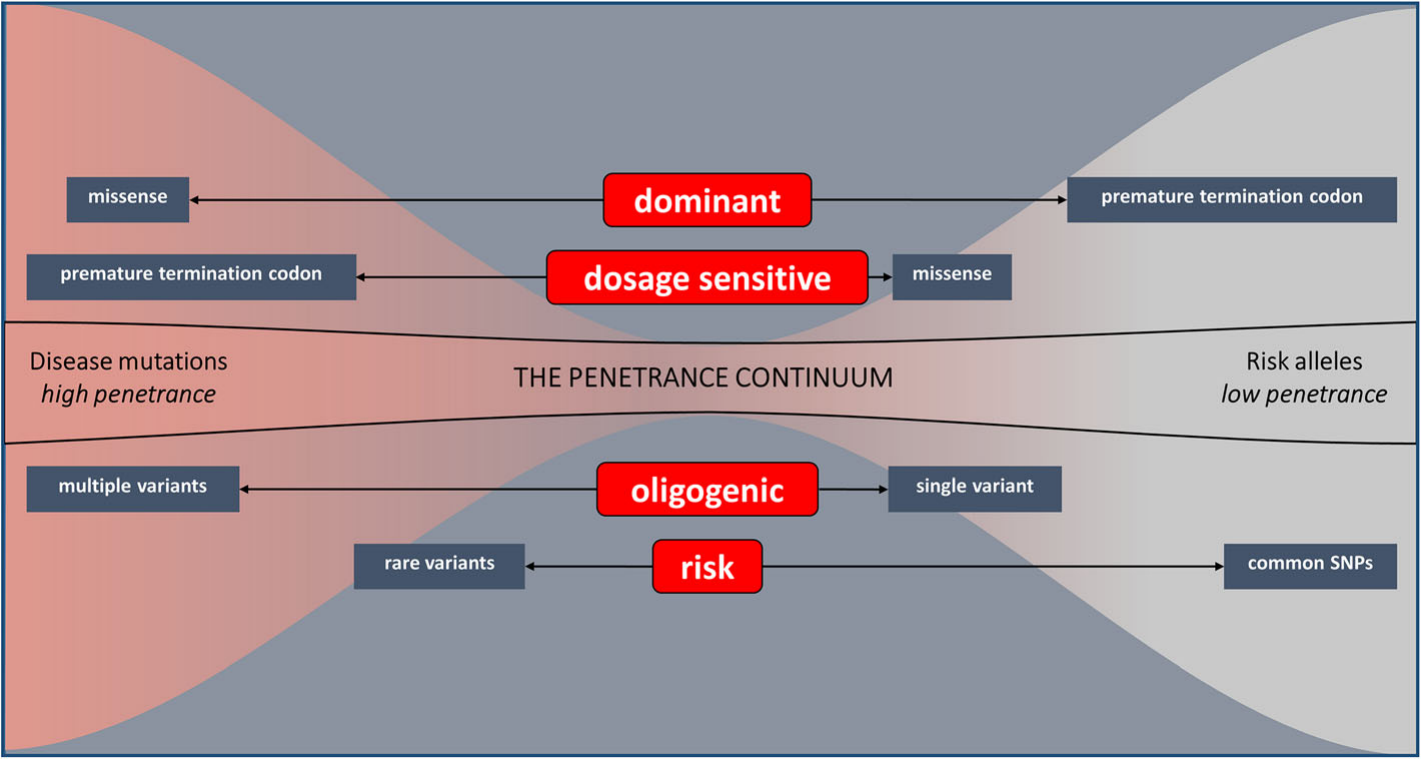
The figure illustrates the penetrance continuum of disease mutations at the extremes, high penetrance (left) and low penetrance (right). The missense mutations in autosomal dominant disease genes (e.g., PSEN1) are highly pathogenic (extreme left) while the role of rare PTC variants needs to be addressed (extreme right). In dosage-sensitive genes (e.g., GRN), PTC mutations are highly pathogenic (extreme left), but rare missense mutations have variable effects on protein function (right). Oligogenic inheritance might explain the reduced penetrance of some pathogenic mutations, in both dominant and dosage-sensitive genes, since one single variant is not penetrant enough to cause the disease on itself. Combinations of multiple rare variants in disease genes increase the effect on gene expression and disease penetrance (extreme left). In risk genes (e.g., ABCA7), common rare single-nucleotide polymorphisms (SNPs) result in a modest increase of disease risk (extreme right), while rare variants can be highly pathogenic and resemble autosomal dominant inheritance in families (left)

We speculate that rare variants can use different modes of action to contribute to NBD and report some key examples of rare variants in known causal and risk genes that recapitulate these modes of action. In-depth NGS re-sequencing of known causal and GWAS risk genes in large study populations led to the detection of huge numbers of rare variants. There is increasing evidence that rare variants of high to intermediate penetrance, and common risk variants with minor effect, are contributing to NBD genetic complexity via different modes of action (Fig. 1).

Haploinsufficiency caused by LOF due to a PTC mutation resulting from a frameshift, nonsense, or splice site mutation is the pathogenic mechanism associated with *GRN* in FTD and with *TBK1* in ALS, FTD, and ALS plus FTD. In addition to PTC mutations, genetic studies reported rare missense variants in both *GRN* and *TBK1*, but their contribution to disease is not yet clear due to limited functional data. In cerebrospinal fluid (CSF) and plasma or serum of *GRN* PTC carriers, GRN levels are reduced to 50%, in line with haploinsufficiency of pathogenic *GRN* PTC mutations [81]. Additionally, few *GRN* missense variants also reduced GRN to intermediate levels in  *GRN* PTC carriers and control individuals [81]. The observation of *GRN* missense variants highlights their possible involvement in disease pathogenesis. Further, in vitro studies showed that the *GRN* p.P248L and p.R432C variants affect GRN secretion and degradation [82, 83]. A direct link between disease and biological mechanisms triggered by missense variants is yet to be demonstrated. Deciphering the role of GRN in FTD pathogenesis can reveal additional insight into the potential pathogenicity of *GRN* missense mutations. Similarly, rare missense mutations in *TBK1* are identified in both FTD, FTD plus ALS, and ALS patients and healthy controls [84, 85]. In vitro studies demonstrated that specific *TBK1* missense mutations affect TBK1 homodimerization, which is essential for TBK1 activation and function, for its kinase activity and its interaction with OPTN [85, 86]. *GRN* and *TBK1* missense mutations were also observed in early-onset [87] and late-onset AD [88] patients, but they were not functionally investigated in vitro or in vivo in relation to AD. These missense variants are important to investigate because they may impair the normal protein function to some extent. Some missense mutations are present only in healthy controls [89] and are a powerful tool to investigate protective biological processes that might slow disease progression and in developing new therapeutic strategies.

Rare PTC variants are also present in other NBD genes, but the majority are not characterized or incorrectly interpreted [60]. For example, in the AD gene *PSEN2*, four potential frameshift mutations were observed in patients [60, 90, 91], while only one is labeled pathogenic in the Alzforum Database, nonetheless, it lacks functional investigation [90]. One frameshift mutation, p.G359Lfs89*, showed a nearly 50% reduction of PSEN2 protein [60]. The same study reported two *PSEN2* frameshift mutations, one in an ALS and one in a FTD patient, two different clinical NBD phenotypes, suggesting that the frameshift variants are unlikely pathogenic. LOF mechanism is proposed for *PSEN1* and *PSEN2* mutations in AD, showing that total *PSEN1* and *PSEN2* LOF in mouse brain caused progressive cognitive decline and neurodegeneration [92]. For instance, the *PSEN1* p.L435F and p.C410Y mutations produced almost complete loss of γ-secretase-dependent processing of APP, without Aβ generation [92]. Newer evidence opposed the hypothesis of LOF for *PSEN1* and *PSEN2* mutations [93]. It is shown that familial *PSEN1* mutations affect the endoproteolytic activities of γ-secretase in a variable way, though no examples of full inactivation have been reported [93]. Patients carrying a familial *PSEN1* mutation express one normal allele of *PSEN1* and two normal alleles of *PSEN2* and can compensate for the loss of normal activity of the mutated allele [93]. PSEN1 and PSEN2 are the catalytic subunits of the γ-secretase complex, mainly involved in APP cleavage. Other functions are proposed, for example, PSEN2 selectively cleaves late endosomal/lysosomal localized substrates [94]. The contribution of PTC mutations in dominant genes warrants further investigation since they might interfere with secondary functions of the encoded proteins.

## Variable expression: age-related reduced penetrance and genetic modifiers

To ensure accurate NBD genetic diagnoses, identification of pathogenic mutations causing disease is of major importance, but a few features may complicate their interpretation. Carriers of the same pathogenic mutation often show a wide range of variation in disease onset age and in clinical phenotype [68]. Some pathogenic mutations are also present in asymptomatic carriers aged above the average onset age in the family and in healthy participants. These observations are challenging our interpretation of rare variants and their role in disease, pointing at how crucial it is to decipher their pathological effects. For example, the *APP* p.A713T mutation is identified in 24 carriers, including asymptomatic carriers, of 11 Italian families who present with highly variable onset ages ranging from 52 to 82 years [60, 95]. Variable onset ages are also observed in members of the same family and unrelated carriers of the same *PSEN2* mutation, p.A85V, p.N141I (Volga German mutation), p.M239V, with a difference of onset age of ≥20 years [96]. In the Volga German AD families, there is evidence that the variable onset age might be explained by the influence of apolipoprotein E (*APOE*) ε4 alleles, a major risk factor for AD [97].

In the world’s largest autosomal dominant AD pedigree of about 5000 living members, spanning five to seven generations and carrying the pathogenic *PSEN1* p.E280A mutation, one carrier had no signs of cognitive impairment until the seventies, three decades after the expected onset age. This carrier had high levels of amyloid β in the brain and was homozygous for the *APOE* ε3 Christchurch (p.R136S) mutation [98]. These findings demonstrate how strong a genetic variant can modify disease onset also in the presence of highly penetrant pathogenic mutations, supporting the role of *APOE* genotypes in AD. Genetic modifiers are proposed to be associated with onset age variability in the Belgian FTD founder pedigree, segregating the *GRN* IVS1+5G>C LOF mutation [68]. The transmembrane protein 106B gene (*TMEM106B*) genotypes are shown to explain part of onset age variability in carriers of different PTC mutations in *GRN* leading to LOF [99].

In PD, the p.G2019S missense mutation in *LRRK2* is the most common missense mutation and one of few *LRRK2* missense mutations considered to be pathogenic based on co-segregation with disease [100]. The *LRRK2* p.G2019S carriers have an onset age range from 59 to 79 years, though some carriers remain asymptomatic till 80 or 90 years [101]. Several studies searched modifiers for *LRRK2* p.G20129S and identified dynamin-3 (*DNM3*) as a candidate, however with significant heterogeneity across studies [102].

The interpretation of pathogenic mutations is unfortunately not always straightforward. Some of the mutations show variable penetrance. The presence of genetic modifiers is a plausible explanation for the reduced penetrance of these mutations. Alternatively, the effects of these pathogenic mutations may not be sufficient to trigger disease on their own, since they may need additional rare mutations in the same or other genes [103].

Rare variants can function as genetic modifiers influencing onset age, clinical phenotype, and disease penetrance, explaining part of the frequently observed variability among unrelated patients and affected relatives in one family [68]. It is possible to identify rare variants in disease genes that are protective, for example, the Icelandic *APP* mutation p.A673T [89]. This *APP* mutation is within the codon of the pathogenic *APP* p.A673V mutation. In Iceland, this protective p.A673T mutation is five times more frequent in healthy people than in AD patients and is associated with a minimal deposition of Aβ in the brain. Rare variants might influence disease onset age in a way comparable to the allelic effects of the *APOE* ε4 allele [104]. Carriers of one or two *APOE* ε4 alleles have a 3- to 15-fold higher risk of developing late-onset AD [105] and are higher in early-onset AD patients (age at onset < 65 years) with a positive family history [106]. Studies have suggested a similar role for the sortilin-related receptor 1 gene (*SORL1*). Some studies reported that rare *SORL1* PTC variants are associated with a fivefold increased risk for early-onset AD, suggesting a comparable risk effect for AD as for carriers of one *APOE* ε4 allele [107]. Identifying risk alleles and modifiers and understanding the role of rare VUS can be relevant to develop effective disease-modifying therapies. Several risk genes have known druggable properties (e.g., sialic acid-binding Ig-like lectin (CD33) in AD [108]) and a translational potential to be targeted and to modify the phenotype, not only in patients but also in individuals at risk. This can help in the selection of patients for clinical trials. For instance, in AD, polygenic risk scores can be calculated to identify individuals at high risk who may benefit from specific therapies [107].

Observations of rare variants in multiple genes belonging to the same or similar biological pathway(s) have led to the concept of oligogenic inheritance to explain the complexity of NBD [103]. For many years, WES and WGS allowed the simultaneous analysis of multiple genes and the identification of multiple variants.

In ALS patients, carrying a pathogenic repeat expansion in the chromosome 9 open reading frame 72 gene (*C9orf72*), the concurrence of multiple variants in several ALS-associated genes is documented [103]. This explains in part why there are asymptomatic carriers of pathogenic *C9orf72* expansions in ALS families [103] and might suggest that other mutated genes may be needed to fully express the disease. In the pathogenic *C9orf72* expanded repeat allele, it is not easy to determine the exact number of repeats which play a role by themselves.

Different studies have shown that in ALS patients and families, carrying two or more mutations in ALS-associated genes [103], some patients are developing the disease 10 years earlier than patients carrying a single ALS gene mutation [109]. This oligogenic concept is extendable to FTD. *GRN* PTC mutation carriers were described to carry an additional mutation in TAR DNA-binding protein (*TARDBP*) or a pathogenic *C9orf72* expansion [68]. The *MAPT* p.A152T mutation is unique in individuals in the Basque country and was found in 71% of FTD patients carrying the pathogenic *GRN* c.709-1G>A mutation [110]. In AD, the presence of more than one variant in causal genes in the same patient carrier has been observed. In a Belgian AD cohort, a patient with a pathological AD diagnosis carried both the known *PSEN1* p.G183V variant and the novel *PSEN1* p.P49L [111]. Another AD patient was reported carrying the VUS *PSEN1* p.P355S and *APP* p.G625_S628del [111]. However, since the number of identified double mutation carriers in FTD and AD is limited, the role of these double mutations remains unclear. In PD, there is also evidence that multiple rare variants in causal genes could influence disease, as PD patients, with more than one mutation in PD genes, have an onset age lower than patients carrying only one pathogenic PD mutation [39]. The most frequently reported double mutations are *LRRK2* p.Gly2019Ser together with homozygous *PARK2* mutations [39]. A study investigated oligogenic inheritance by performing WES in 980 neuropathologically characterized human brains from AD, PD, and FTD-ALS patients and age-matched controls [112]. The authors identified in FTD-ALS, AD, and PD, oligogenic cases defined by the presence of more than one variant in the list of NBD genes selected for the study, with minor allele frequency below 1% in the Exome Aggregation Consortium (ExAc) database [112]. The impact of oligogenic mutations on disease expression is currently unclear due to the limited number of oligogenic mutation carriers identified so far and the lack of large families to investigate co-segregation with disease. Deciphering the contribution of rare variants to disease is therefore essential to understand these disorders.

GWAS revealed a wealth of risk genes in NBD. In these risk genes, the variants explaining the association have often remained elusive. Post-GWAS studies aim to decipher the functional variants in these risk loci, but they also reveal a more complex genetic picture. In the ATP-binding cassette sub-family A member 7gene (*ABCA7*), both common and rare variants are reported to affect AD risk [113]. Rare *ABCA7* PTC variants of intermediate to high penetrance are observed with a 1.5–4-fold increased frequency in AD patients across populations [113]. Transcripts containing PTC variants are degraded by nonsense-mediated mRNA decay to avoid the formation of truncated proteins, resulting in LOF. A few pedigrees have been reported in which *ABCA7* PTC variants mimicking co-segregating with disease in an autosomal dominant inheritance [114, 115]. For this reason, it is still debatable to consider these variants as high-penetrant mutations like *APP*, *PSEN1*, and *PSEN2* mutations in AD. A few rare variants in GWAS-associated genes are high-penetrant mutations, compared to the common single nucleotide polymorphisms (SNPs) detected in the association studies. Functional characterization of these rare variants is of major importance, because they exert a pathogenic effect on disease progression. Importantly, patients carrying these variants need genetic counseling as well as effective treatments.

## The potential of additional omics tools to provide insights into disease etiology

Advances in other omics tools are contributing to increase understanding of unknown genetic causes, post-genomic effects, and molecular pathways of NBD. Among them, long-read sequencing (e.g., Oxford Nanopore Technology) enables the detection of structural variants [116], short tandem repeats (STR) [117], and variable number of tandem repeats (VNTRs) [118]. High-throughput RNA sequencing (RNA-seq) transcriptomics (e.g., single-cell and single-nuclei RNA-seq) can identify expression signatures potentially associated with disease pathology, providing important insights into potential subpopulations of cells directly involved in disease [119]. A study showed that the brain of carriers of rare pathogenic *APP*, *PSEN1*, or *PSEN2* mutations presented with lower neuron and higher astrocyte relative proportions compared to sporadic AD patients [120]. Similarly, the *APOE* ε4 allele also showed decreased neuronal and increased astrocyte relative proportions compared to AD non-carriers, while carriers of rare *TREM2* risk variants showed a lower degree of neuronal loss [120]. Proteomics approaches (e.g., spatial proteomics) also contributed to the unraveling of NBD pathogenesis, enabling localizations of proteins and their dynamics at the subcellular level [121]. Epigenetic changes also implicated in NBD pathogenesis have shown an increased methylation state in the promoter region of *C9orf72* repeat expansion carriers [122]. Evidence suggests that metabolomic perturbations in different pathways may mediate the occurrence of NBD, as demonstrated by one of the largest metabolomics studies conducted by researchers from the Alzheimer’s Disease Metabolomics Consortium [123]. These achievements demonstrate the potential of these alternative omics technologies to reveal complex events in relation to NBD.

## Conclusions and future directions

NBD are devastating disorders with yet no current effective treatments. Advances in omics technologies facilitated an increased knowledge of the biological mechanisms underlying CNS neurodegeneration, based on the identification of novel genes and specific pathways contributing to NBD pathophysiology. These advances also increased the detection of rare VUS, of which functional analysis did not keep pace with the development of these methodologies. Understanding the post-genomic consequences of rare variants has direct implications in clinical practice. WGS is predicted to convert to the standard diagnostic tool in medical genetic testing within 5 years [124]. Considering the complexity of NBD, a profound understanding of the role of rare variants will be essential for the design of clinical trials, identifying people at high risk, personalized prevention, and treatment. Recent work demonstrated the potential of patient-derived iPSCs, in combination with genome editing technology and 3D brain organoids, in recapitulating the NBD phenotypes, which are presenting powerful tools for rare variant interpretation. The data generated from WES and WGS, in combination with the information provided by transcriptomics, proteomics, epigenomics, and metabolomics, will expand our understanding of the post-genomic effects of rare genetic variants and the disrupted pathways in NBD. Rare genetic variants in disease genes have received increased attention and their functional interpretation will provide a better understanding of disease pathogenesis, improvement of genetic diagnostic testing, clinical diagnosis, and development of therapeutics for personalized medicine in the future.

## Data Availability

Not applicable.

## Abbreviations

3D: Three-dimensional
ABCA7: ATP-binding cassette sub-family A member 7
ABI3: ABI family member 3 (*ABI3*)
*ACE*: Angiotensin I-converting enzyme
*ACMSD*: Aminocarboxymuconate semialdehyde decarboxylase
AD: Alzheimer’s disease
*ADAM10*: ADAM metallopeptidase domain 10
*ADAMTS1*: ADAM metallopeptidase with thrombospondin type 1 motif 1
ADSP: Alzheimer Disease Sequencing Project
ALS: Amyotrophic lateral sclerosis
*APOE*: Apolipoprotein E
*APP*: Amyloid precursor protein
*ASXL3*: ASXL transcriptional regulator 3
*ATP10B*: ATPase phospholipid transporting 10B
Aβ: Amyloid beta
*BCKDK*: Branched chain ketoacid dehydrogenase kinase
*BIN1*: Bridging integrator 1
*BRIP1*: BRCA1-interacting protein C-terminal helicase 1
*BST1*: Bone marrow stromal cell antigen 1
*BTNL2*: Butyrophilin like 2
*C21orf2*: Chromosome 21 open reading frame 2
*C4orf27*: Chromosome 4 open reading frame 27
*C5orf24*: Chromosome 5 open reading frame 24
*C9orf72*: Chromosome 9 open reading frame 72
*CAB39L*: Calcium-binding protein 39 like
*CASS4*: Cas scaffold protein family member 4
*CCDC62*: Coiled-coil domain containing 62
*CD19*: CD19 molecule
*CD2AP*: CD2-associated protein
*CD3*3: Sialic acid-binding Ig-like lectin 3
*CELF1*: CUGBP Elav-like family member 1
*CHRNB1*: Cholinergic receptor nicotinic beta 1 subunit
*CLCN3*: Chloride voltage-gated channel 3
*CLU*: Clusterin
CNS: Central nervous system
*CR1*: Complement C3b/C4b receptor 1
*CRLS1*: Cardiolipin synthase 1
CSF: Cerebrospinal fluid
*CTSC*: Cathepsin C
*CYLD*: CYLD lysine 63 deubiquitinase
*DDRGK1*: DDRGK domain containing 1
*DGKQ*: Diacylglycerol kinase theta
DIAN-TU: Dominantly Inherited Alzheimer Network Trials Unit
*DNAH17*: Dynein axonemal heavy chain 17
DNM3: Dynamin-3
*DPP6*: Dipeptidyl peptidase like 6
*DSG2*: Desmoglein 2
*DYRK1A*: Dual-specificity tyrosine phosphorylation regulated kinase 1A
*EPHA1*: EPH receptor A1
ExAc: Exome Aggregation Consortium
*FAM171A2*: Family with sequence similarity 171 member A2
*FAM47E*: Family with sequence similarity 47 member E
*FAM49B*: CYFIP-related Rac1 interactor B
*FBRSL1*: Fibrosin like 1
*FCGR2A*: Fc fragment of IgG receptor IIa
*FERMT2*: Fermitin family member 2
*FGF20*: Fibroblast growth factor 20
*FGGY*: FGGY carbohydrate kinase domain containing
FTD: Frontotemporal dementia
*FUS*: Fused in sarcoma
*FYN*: FYN proto-oncogene, Src family tyrosine kinase
*GAK*: Cyclin G-associated kinase
*GBA*: Beta acid glucosidase
*GBF1*: Golgi brefeldin A-resistant guanine nucleotide exchange factor 1
*GPNMB*: Glycoprotein nmb
*GRN*: Granulin
GWAS: Genome-wide association studies
*HIP1*: Huntingtin-interacting protein 1
*HLA-DQA2*: Major histocompatibility complex, class II, DQ alpha 2
*HLA-DQB1*: Major histocompatibility complex, class II, DQ beta 1
*HLA-DRA*: Major histocompatibility complex, class II, DR alpha
*HLA-DRB1*: Major histocompatibility complex, class II, DR beta 1
*HLA-DRB5*: Major histocompatibility complex, class II, DR beta 5
hPSCs: Human pluripotent stem cells
IFGC: International Frontotemporal Dementia Genomics Consortium
IGAP: International Genomics of Alzheimer’s Project
*IMMP2L*: Inner mitochondrial membrane peptidase subunit 2
*INPP5D*: Inositol polyphosphate-5-phosphatase D
*INPP5F*: Inositol polyphosphate-5-phosphatase F
IPDGC: International Parkinson’s disease Genomics Consortium
iPSCs: Induced pluripotent stem cells
*IQC*K: IQ motif containing K
*IRF2*: Interferon regulatory factor 2
*ITPR2*: Inositol 1,4,5-trisphosphate receptor type 2
*KAT8*: Lysine acetyltransferase 8
*KCNIP3*: Potassium voltage-gated channel interacting protein 3
*KCNS3*: Potassium voltage-gated channel modifier subfamily S member 3
*KIF5A*: Kinesin family member 5A
*KPNA1*: Karyopherin subunit alpha 1
*LAMP3*: Lysosomal-associated membrane protein 3
*LCORL*: Ligand-dependent nuclear receptor corepressor like
*LINC00693*: Long intergenic non-protein coding RNA 693
*LOC101927354*: na
*LOC101927815*: na
LOF: Loss-of-function
*LRRK2*: Leucine-rich repeat kinase 2
*MAPT*: Microtubule-associated protein tau
*MBNL2*: Muscleblind like splicing regulator 2
*MCCC1*: Methylcrotonoyl-CoA carboxylase 1
*MED12L*: Mediator complex subunit 12 L
*MEF2C*: Myocyte enhancer factor 2C
*MEX3C*: mex-3 RNA binding family member C
*MIPOL1*: Mirror image polydactyly 1
*MIR548*AP: MicroRNA 548ap
*MOBP*: Myelin-associated oligodendrocyte basic protein
*MS4A6A*: Membrane spanning 4-domains A6A
NBD: Neurodegenerative brain diseases
NGS: Next-generation sequencing
*NME8*: NME/NM23 family member 8
*NOD2*: Nucleotide-binding oligomerization domain containing 2
*NUCKS*: Nuclear casein kinase and cyclin-dependent kinase substrate 1
*OLFM1*: Olfactomedin 1
*OPTN*: Optineurin
*PAM*: Peptidylglycine alpha-amidating monooxygenase
*PARK2*: Parkin 2
PD: Parkinson’s disease
*PICALM*: Phosphatidylinositol-binding clathrin assembly protein
*PINK1*: PTEN-induced kinase 1
*PLCG2*: Phospholipase C Gamma 2
*PRNP*: Prion protein
*PSEN1*: Presenilin 1
*PSEN2*: Presenilin 2
PTC: Premature termination codon
*PTK2B*: Protein tyrosine kinase 2 beta
*RAB29*: RAB29, member RAS oncogene family
*RAB38*: RAB38, member RAS oncogene family
*RAI1*: 1-Retinoic acid induced
*RERG*: RAS-like estrogen-regulated growth inhibitor
*RIMS1*: Regulating synaptic membrane exocytosis 1
*RIT2*: Ras-like without CAAX 2
RNA-seq: RNA sequencing
*RNF141*: Ring finger protein 141
*RPS12*: Ribosomal protein S12
*RPS6KL1*: Ribosomal protein S6 kinase-like 1
*SARM1*: Sterile alpha and TIR motif containing 1
*SCAF11*: SR-related CTD-associated factor 11
*SCARB2*: Scavenger receptor class B member 2
*SCFD1*: sec1 family domain containing 1
*SIPA1L*2: Signal-induced proliferation-associated 1 like 2
*SLC24A4*: Solute carrier family 24 member 4
*SNCA*: α-Synuclein
SNPs: Single-nucleotide polymorphism
*SOD1*: Cu/Zn superoxide dismutase
*SORL1*: Sortilin-related receptor 1
*SPTSSB*: Serine palmitoyltransferase small subunit B
*SQSTM1*: Sequestosome 1
*SREBF1*: Sterol regulatory element-binding transcription factor 1
*STBD1*: Starch-binding domain 1
*STK39*: Serine threonine kinase 39
STR: Short tandem repeat
*STX1B*: Syntaxin 1B
*SYT11*: Synaptotagmin XI
TARDBP: TAR DNA-binding protein
*TARDBP*: Transactive response DNA-binding protein
*TBK1*: TANK-binding kinase 1 gene
TDP-43: TAR DNA-binding protein 43
*TIA1*: Cytotoxic granule-associated RNA-binding protein
*TMEM106B*: Transmembrane protein 106B
*TMEM163*: Transmembrane protein 163
*TMEM175*: Transmembrane protein 175
*TREM2*: Triggering receptor expressed on myeloid cells
*TRIM40*: Tripartite motif containing 40
*UBAP2*: Ubiquitin-associated protein 2
*UBTF*: Upstream binding transcription factor
*UNC13A*: unc-13 homolog A
*VAMP4*: Vesicle-associated membrane protein 4
*VCP*: Valosin-containing protein gene
VNTR: Variable number of tandem repeats
*VPS13C*: Vacuolar protein sorting 13 homolog C
VUS: Variants of uncertain significance
WES: Whole-exome sequencing
WGS: Whole-genome sequencing
*WWOX*: WW domain-containing oxidoreductase
*ZCWPW1*: Zinc finger CW-type and PWWP domain containing
